# A Case Report of an Isolated Dislocation of the Scaphoid in a Lesser Arc Injury and a Review of the Literature

**DOI:** 10.1155/2018/9591502

**Published:** 2018-06-11

**Authors:** Ryan Sefcik, Kyle Andrews, Jacob Stirton, Justin Lea, Mina Tanios, Martin Skie

**Affiliations:** Department of Orthopaedic Surgery, University of Toledo College of Medicine and Life Sciences, Toledo, OH, USA

## Abstract

Isolated dislocations of the scaphoid are extremely uncommon injuries and are often associated with significant ligamentous failures. Since scaphoid dislocations typically present with associated carpal fractures, few cases of isolated dislocations of the scaphoid exist in the literature. The proposed treatment options in the literature range from closed reduction and casting to open reduction and internal fixation. We present the case of a 41-year-old male with an isolated scaphoid dislocation in whom open reduction and internal fixation was performed with K-wires. At five months follow-up, the patient had returned to work and all desired activities.

## 1. Introduction

Isolated scaphoid dislocations are extremely rare injuries and only individual case reports have been published in the current literature. In 1930, Higgs [1] became the first to report on isolated scaphoid dislocations. Many years have passed with relatively few reports detailing this injury. Leung et al. [2] classified pure scaphoid dislocations as simple or complex and partial or total. They also went on to report that the proximal pole has been observed to displace in the volar, dorsal, or radial directions.

The exact mechanism of the injury is unknown; however, previous reports have suggested that high-energy motor vehicle accidents with forced wrist dorsiflexion of the hand in ulnar deviation may result in the scaphoid leaving its fossa [3]. Of the few reported cases, treatment strategies have included closed reduction with casting, closed reduction and percutaneous pinning using Kirschner wires (K-wire), open reduction and pinning, and open reduction and pinning with ligament repair [3–5].

This case report details our experience in treating an isolated scaphoid dislocation in a 41-year-old man following a motorcycle accident. Open reduction with K-wire fixation was performed after a failed closed reduction in the emergency department. Since few case reports exist in the literature and present varying strategies, our case report may be a valuable resource for future diagnosis and treatment of similar injuries.

## 2. Case Report

**Figure 1:**
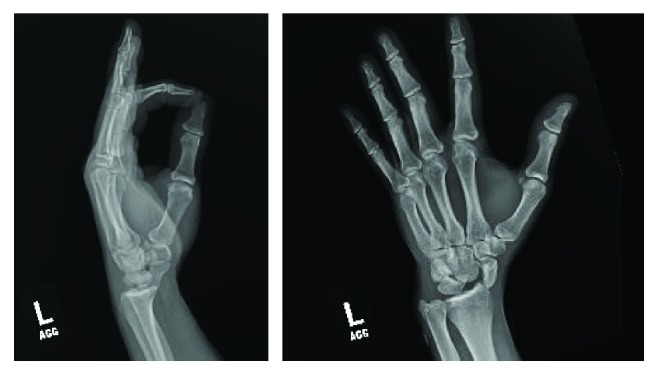
Presenting plain films demonstrating volar scaphoid dislocation.

**Figure 2:**
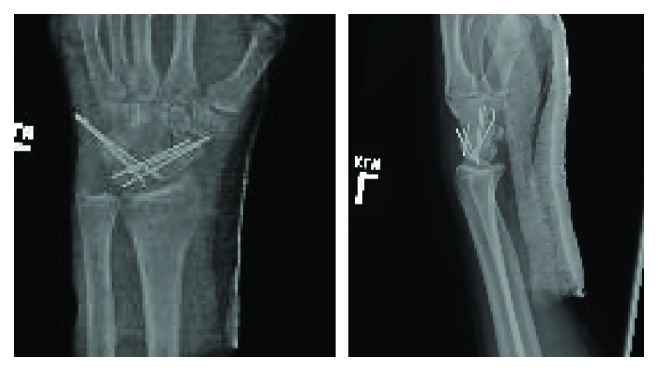
Postoperative radiographs.

**Figure 3:**
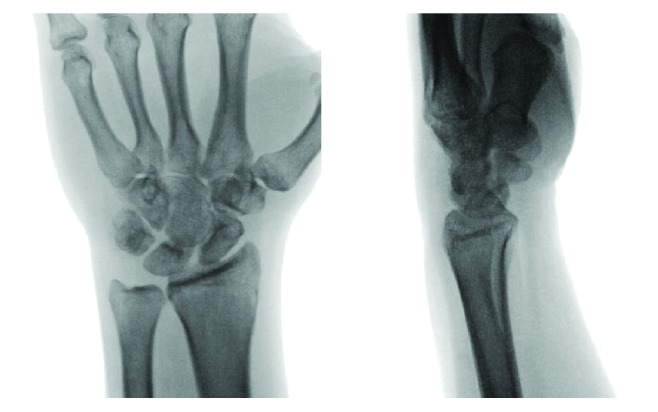
Plain films at the five months postoperative visit.

A 41-year-old man presented to the emergency department following an automotive accident where he was thrown from his motorcycle traveling approximately 35 mph. The patient denied loss-of-consciousness and chiefly complained of left wrist pain at presentation with exam demonstrating tenderness and swelling. Radiographs (Figure 1) revealed a volar dislocation and rotatory deformity of the proximal pole of the scaphoid. The distal pole remained properly located, articulating with the trapezium and trapezoid. No other injuries were identified. Closed reduction failed in the emergency department; therefore, the patient elected to proceed with operative management. A dorsal approach was used to access the radiocarpal joint. The scapholunate joint was completely disrupted and the lunotriquetral joint was found to be unstable as well. The distal pole of the scaphoid appeared to be appropriately located without ligamentous disruption. No fractures or chondral injuries were seen. Three 0.045-inch K-wires were placed into the proximal pole and used as a joystick in concert with dorsally directed manual pressure over the distal pole to reduce the scaphoid dislocation. These were then advanced across the scapholunate articulation to hold the reduction. Three more K-wires were passed across the lunotriquetral joint to address the instability. The distal pole was once again examined but did not demonstrate any instability. The capsule was closed with 0 VICRYL suture, and the K-wires were cut just below the skin. The subcutaneous layer was closed with buried 2–0 VICRYL suture, the skin was closed with 4–0 NOVAFIL suture, and the wrist was splinted (Figure 2). The patient did well postoperatively and was brought back to the operating room eight weeks later for hardware removal. He went on to heal well and regained his wrist range of motion with occupational therapy. At most recent follow-up (Figure 3), five months since injury, the patient had no complaints and had returned to work and all desired activities.

## 3. Discussion

Substantial force is required to disrupt the scaphoid from its fossa which more commonly fractures the radial styloid or the scaphoid waist [6]. As a result, isolated scaphoid dislocations without secondary fractures, such as that in the presented case, are especially uncommon.

The severity of the injury is largely dependent on the number of ligaments disrupted and it is postulated that the order of ligamentous failure begins with the radioscaphocapitate and scapholunate ligaments followed by the radiolunate and scaphotrapezial ligaments [7]. Isolated scaphoid dislocations were initially divided into simple or complex based on distal carpal row integrity [8]. More recently, Leung et al. further divided the classification of these injuries into the following: primary versus secondary, simple versus complex, partial versus total, and the direction of the dislocation [2]. Primary dislocations are dislocations that result directly from the injury and secondary dislocations are persisting dislocations following failed closed reduction. Simple dislocations involve only the scapholunate and radioscaphoid while complex types have distal carpal row involvement. In partial dislocations, the proximal pole of the scaphoid is found outside of the fossa but the soft tissue attachments to the distal portion are intact. Complete dislocations refer to injuries in which the dislocated scaphoid lacks any soft tissue attachments.

Chloros et al. created an algorithm for treating isolated carpal dislocations [3]. They recommended open reduction and K-wire fixation for complex dislocations, simple dislocations with palmar-ulnar dislocations, and simple dislocations with delayed treatment. Closed reduction and casting may be satisfactory for simple dislocations in which there was no delay in treatment. However, if closed reduction fails and a secondary dislocation remains, the most appropriate treatment option would be open reduction and K-wire fixation.

Occasionally, the diagnosis of isolated scaphoid dislocations may be missed; however, standard radiographs were sufficient to diagnose in this case [9]. Few complications exist with isolated scaphoid dislocations. One possible complication is avascular necrosis of the scaphoid. This complication has only been reported once in the literature [7]. It appears that undisturbed intraosseous channels within the scaphoid bone allow for rapid revascularization from the surrounding soft tissue. Other possible complications include posttraumatic arthritis and carpal instability [10]. These are thought to be more closely related to neglected cases, those in which there was a delay in treatment.

## 4. Conclusion

Isolated scaphoid dislocations are uncommon injuries and frequently missed. The prognosis of isolated dislocations of the scaphoid is typically good when treated early and appropriately. Open reduction and percutaneous pinning is a sufficient and effective treatment for isolated scaphoid dislocations, especially in complex cases and secondary dislocations following failed closed reduction. Complications are uncommon, and most patients will return to their previous activities following proper treatment.
